# Modified Cangfu Daotan decoction ameliorates polycystic ovary syndrome with insulin resistance *via* NF-κB/LCN-2 signaling pathway in inflammatory microenvironment

**DOI:** 10.3389/fendo.2022.975724

**Published:** 2022-11-07

**Authors:** Shuowen Liu, Yao Zhang, Fang Yang, Jingna Gu, Ruyue Zhang, Yingying Kuang, Wantong Mai, Chengbo Zheng, Yang Yu, Ruling Lu, Lei Zeng, Hongying Cao, Yongling Long

**Affiliations:** ^1^ School of Pharmaceutical Science, Guangzhou University of Chinese Medicine, Guangzhou, Guangdong, China; ^2^ Department of Gynecology, the First Affiliated Hospital of Guangzhou University of Chinese Medicine, Guangzhou, China

**Keywords:** Lcn-2, NF-kB signaling pathway, INSR/IRS-1/GLUT4, PCOS-IR, Modified Cangfu Daotan decoction

## Abstract

This study explored the possible connection between the insulin resistance-targeting protein adipokine lipocalin-2 (LCN-2) and NF-κB signaling pathway in the inflammatory microenvironment in PCOS-IR model rats to determine the pharmacological mechanism of modified Cangfu Daotan decoction (MCDD) intervention for PCOS-IR. We used a high-fat diet (42 days) combined with letrozole (1 mg/kg/day, 42 days) to establish a PCOS-IR rat model. From the third week after modeling, the rats were given continuous administration of MCDD (high dose with 31.68 g/kg, medium dose with 15.84 g/kg, and low dose with 7.92 g/kg) for 28 days. Serum, ovarian tissue, liver, and adipose tissue were collected after the last gavage. Enzyme-linked immunosorbent assay, hematoxylin-eosin (HE) staining, Masson staining, qRT-PCR, and Western blot experiments were performed to detect various indicators. Our results showed that MCDD could reduce body weight and abdominal fat weight; restore normal estrous cycle and ovarian function; alleviate fatty liver; regulate HOMA-IR and OGTT index; reduce serum inflammatory factor levels, LCN-2 level, and gene expression; and regulate the insulin signal transduction and NF-κB pathways in PCOS-IR rats. Thus, MCDD may play a role in improving ovarian function in PCOS-IR rats by downregulating NF-κB/LCN-2 proteins and upregulating the gene expression of *Insr/Irs-1/Glut4* in the insulin signaling pathway in the inflammatory environment.

## Introduction

Polycystic ovary syndrome (PCOS), a complex endocrine disorder with reproductive and metabolic heterogeneity, is the leading cause of female endometrial hyperplasia and infertility worldwide. The global prevalence of PCOS is 8%–13% (1, 2). PCOS also increases the risk of cardiovascular disease, obesity, impaired glucose tolerance, type 2 diabetes, dyslipidemia, hypertension, and a series of metabolic syndromes in women. Obesity is a common problem in patients with PCOS, of which the obesity rate varies between 50% and 80% depending on the population studied (2). As a result of the influence of genetic, environmental, and epigenetic factors, the pathogenesis of PCOS is complex, and the mechanism of action of clinical treatment needs to be further explored. Therefore, the prevention and treatment of PCOS have become one of the public health problems to be solved.

The pathogenesis of PCOS is unclear, and hyperandrogenism (HA) and hyperinsulinemia (HI) are its main pathological features. HA is a major factor driving the development of PCOS features (3). Most women diagnosed with PCOS, especially those with HA phenotype, have the highest incidence of insulin resistance (IR), which is exacerbated by obesity (4). Excess androgen induces IR, and the compensatory elevation of insulin to form HI promotes the deposition of abdominal fat and visceral adipose tissue and further promotes androgen secretion in the ovaries and adrenal glands in women with PCOS. A variety of factors lead to the deficiency of insulin’s ability to promote the uptake and utilization of glucose by organs, tissues, and cells, eventually resulting in IR (5). The interaction between HI and HA caused by IR forms a vicious circle, which further aggravates PCOS (6). Women with clinically severe ovarian dysfunction, in addition to the manifestations of androgen excess, ovulation, and menstrual dysfunction, are accompanied with severe IR (7).

Adipokine lipocalin-2 (LCN-2), also known as neutrophil gelatinase-associated lipocalin (NGAL), is a kind of 25 kDa secreted glycoprotein that plays key roles in regulating body fat mass, IR, and lipid metabolism (7). The mRNA expression of LCN-2 is significantly increased in both adipose and liver tissues in obese and diabetic mice (8). The levels of circulating LCN-2 are positively correlated with obesity, hyperglycemia, hypertriglyceridemia, and IR indices (9–13). The levels of circulating LCN-2 in patients with abnormal glucose tolerance are significantly high (14). Chan found that LCN-2 reduces insulin sensitivity in heart-derived cell lines by inhibiting the insulin-stimulated Akt/p70S6K signaling pathways in a time- and dose-dependent manner (15). All of the above suggest that LCN-2 may be a target of ameliorating IR. However, the role of LCN-2 in the pathogenesis of PCOS-IR remains unclear.

Patients with PCOS are often accompanied with abdominal obesity. Obesity is a low-grade chronic inflammatory state. A number of studies on the molecular mechanism of metabolic syndrome (MetS) have revealed that LCN-2 is closely related to low-grade chronic inflammation. Many adipocyte-derived adipokines such as tumor necrosis factor-α (TNF-α) are involved in the regulation of metabolic homeostasis and display immunomodulatory properties (16). After treatment with TNF-α (100 ng/mL), the expression of LCN-2 in astrocytes was reported to significantly increase (17). IL-17A/TNF-α can activate NF-κB signaling and promote the binding of NF-κB to LCN-2 (18). Inflammatory markers in patients with PCOS, such as CRP, IL-6, IL-18, and TNF-α, sharply increase, and this increase is more significant in patients with PCOS-IR than in the controls (19–21). In the state of low-grade inflammation, inflammatory factors reduce insulin sensitivity and induce IR by interfering with insulin signaling pathways, such as the PI3K/AKT pathway, promoting lipolysis, and reducing peroxidase proliferating receptor γ (PPARγ). Clinically insulin sensitizers metformin and pioglitazone are used to inhibit the activity of NF-κB through the PI3K-MAPK signaling pathway. Therefore, the changes in levels of inflammatory factors play key roles in the treatment of PCOS-IR. Gnanadass emphasized the role of inflammatory markers in ovarian regulation reducing the risk of PCOS (Abraham 22). Sufficient research on the low-grade inflammation of PCOS is lacking.

Evidence-based guidelines suggest that metformin in combination with lifestyle interventions may be the most effective treatment for PCOS. Modern medical treatment often uses metformin combine with oral contraceptives to treat patients of PCOS-IR. Metformin has been shown to help regulate high insulin hematic disease, lower testosterone levels, and control the menstrual cycle of women with PCOS. Metformin can improve the metabolism disorders of PCOS, such as IR and type 2 diabetes, but it can still cause slight effects such as, temporary suppression of appetite, nausea or vomiting, abdominal distension, and diarrhea (3). There is still a great need to develop safe and effective traditional Chinese medicine (TCM) compounds against PCOS-IR. Herbs can be used to treat irregular menstruation, infertility, and IR in PCOS; regulate the menstrual cycle; and reduce luteinizing hormone levels (23–25). After years of experience and innovative development, the advantages of TCM have become prominent. Modified Cangfu Daotan decoction (MCDD) is an empirical prescription for clinical treatment. It invigorates the spleen and eliminates phlegm, tonifies the kidney, and regulates the meridian as the main treatment method, thereby significantly reducing fat and eliminating phlegm in patients with PCOS-IR and significantly improving IR (26–30). However, the specific mechanism underlying the improvement of PCOS-IR by this prescription needs further study.

As a result, we targeted LCN-2 to study the molecular mechanism of MCDD in improving PCOS-IR by regulating NF-κB/LCN-2 signaling in the inflammatory microenvironment. The molecular pharmacological mechanism of MCDD in the treatment of PCOS-IR has great theoretical value and clinical significance.

## Materials and methods

### Animals

Seventy-two six-week-old female SD rats (weighing 150–170 g) were purchased from Guangdong Medical Laboratory Animal Center, production license number: SCXK (Guangdong) 2018-0002. Animals were raised in SPF, Experimental Animal Center, School of Traditional Chinese Medicine, Guangzhou University of Traditional Chinese Medicine Class animal breeding room, license number: SYXK (Guangdong) 2019-0202. The rearing environment was a temperature of 20°C ± 5°C, relative humidity of 40°C to 50°C, and alternating light for 12 h. The experiment was carried out under the management of animal ethics review of Guangzhou University of Chinese Medicine. It was reviewed and approved by the Animal Ethics Review Committee of the School of Chinese Medicine, Guangzhou University of Chinese Medicine.

### Reagents and materials

General control feed (GD 450 J) and high-fat feed (GD 60) were purchased from Guangdong Medical Experimental Animal Center, license number: Guangdong feeding Certificate (2019) 05073. High-fat feed energy with 5.24 kcal/g, and the energy ratio was 60% fat, 20% carbohydrate, and 20% protein. Letrozole (national drug standard H19991001, Jiangsu Hengrui Pharmaceutical Co., Ltd.), sodium carboxymethyl cellulose (Shanghai Aladdin biochemical Technology Co., Ltd.), Roche blood glucose meter and test paper (Roche Diagnostic Co., Ltd.), glucose powder (H51020922, Kangmei Baoning Sichuan Pharmaceutical Co., Ltd.), 4% Lu alkaline methylene blue dye (Beijing Soleibao Technology Co., Ltd.), HE, Masson, and Oil Red O Stain Set were purchased at Wuhan Servicebio Technology CO., Ltd. Positioned optical microscope (Olymplus, Japan) and digital pathological section scanning system (Pannoramic MIDI) were employed. Serum total testosterone (T, RXJ302700R), estradiol (E2, RXJ302812R), luteinizing hormone (LH, RXJ303076R), follicle stimulating hormone (FSH, RX302805R), sex hormone binding protein (SHBG, RX302223R), lipotroponin (LCN-2, RX302066R), IL-1 (RX302881R), IL-1β (RX302869R), IFN-γ (RX302900R), TNF-α (RX302058R), IL-6 (RX302856R) ELISA kit with TCHOL (RXWB0294), TG (RXWB0011), LDL-C (RXSH0759), and HDL-C (RXSH0757) were measured by using automatic biochemical analyzers, and they were all procured from Quanzhou Ruixin Biological Technology Co., Ltd. (Quanzhou, China). Fasting insulin ELISA kit (FINS, CSBE05070r) was from Wuhan Huamei Biological Engineering Co., Ltd. All primers in **Table 1** were synthesized from Sangon Biotech (Shanghai) Co., Ltd. Tissue RNA Purification Kit PLUS Kit (Cat. No.: EZB-RN001-plus), Color Reverse Transcription Kit (Cat. No.: A0010CGQ), and 2x Color SYBR Green qPCR Master Mix (Cat. No.: A0012-R2) were from EZBioscience.

**Table 1:** Primer sequences for qRT PCR analysis.

Gene	Sense primer	Antisense primer
*Insr*	CAGTGTCGTGATCGGAAGTATT	CTGAGGTACTCTGGGTTTGAAG
*Irs-1*	ATGTGGAAATGGCTCGGA	TAAGGCAGCAAAGGGTAGGC
*Glut4*	ACGTTGGTCTCGGTGCTCTTA	ATGGATGGAACCCGCTCCA
*Lcn-2*	GGAATATTCACAGCTACCCTC	TTGTTATCCTTGAGGCCCAG
*Tnf-α*	ATGGGCTCCCTCTCATCAGT	AAATGGCAAATCGGCTGACG
*Nf* **-** *κb*	TGCCGAGTAAACCGGAACTC	CAGCCAGGTCCCGTGAAATA
*Gapdh*	AGACAGCCGCATCTTCTTGT	CTTGCCGTGGGTAGAGTCAT

### PCOS-IR rat model

**Figure 1:**
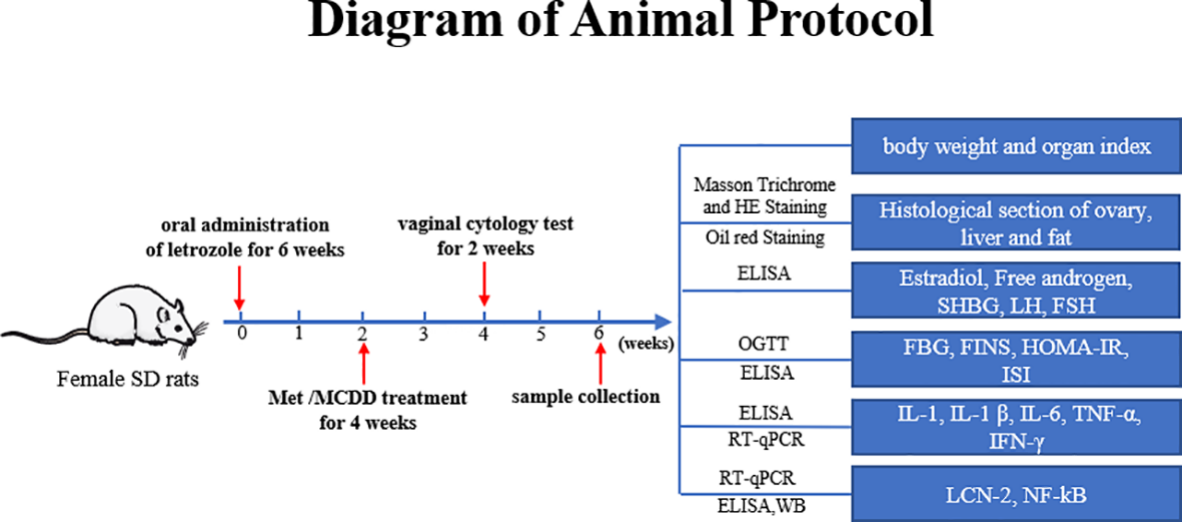
Diagram of Animal Protocol.

After adaptive feeding for 1 week, the rats were randomly divided into two groups: 12 rats in the control group, and the rest were in the model group. The method of vaginal smear was used to observe the successful model, and they were divided into a model group and four drug administration groups. The control group was given a normal control diet for six consecutive weeks, and 0.5% carboxymethyl cellulose sodium (CMC-Na) was given by gavage every day. The remaining 60 rats (model group, metformin group, and high-, medium-, and low-dose MCDD groups) were given high-fat diet for six consecutive weeks and intragastric administration of 1 mg/kg/day letrozole solution dissolved in 0.5% CMC-Na (As shown in **Figure 1**).

### Preparation and treatment of the administration group

MCDD Composition: *Atractylodes lancea* 10 g, *Cyperus rotundus* 10* g*, *Pinellia ternata* 10 g, *Citrus reticulata* 6 g, *Poria cocos* 30 g, *Astragalus membranaceus* 30 g, *Acorus tatarinowii* Schott 10 g, *Dioscorea opposite* 20 g, *Angelica sinensis* 10 g, *Salvia miltiorrhiza* 15* g*, *Epimedium brevicornu* 15 g, and *Gleditsia sinensis* 10 g. These were provided by Guangdong Daxiang Chinese Medicine Pharmaceutical Co., Ltd. Referring to the clinical dose of 176 g/70 kg and the gavage volume of 1 mL/100 g for rats, the equivalent dose for rats was converted, that is, the concentration of decoction was 15.84 g/kg (medium dose), the high dose was twice the equivalent dose, and the low dose was 1/2 the equivalent dose. The intervention treatment started from the third week, and metformin group rats were given 500 mg of metformin solution every day for 4 weeks. Similarly, high-dose (31.68 g/kg), medium-dose (15.84 g/kg, clinical dose 176 g/70 kg), and low-dose (7.92 g/kg) MCDD groups were intervened; the control group and model group were given pure water (As shown in **Figure 1**).

### General condition of rats and vaginal cytology test

The mental state, water intake, defecation, and hair color of the rats in each group were observed daily. In addition, the food intake of the rats in each group was recorded every day, and their body weight was weighed once a week. Vaginal smears were performed at ten o’clock in the morning for 10 consecutive days before rats were sacrificed, and the periodical changes of vaginal exfoliated cells of the rats in each group were observed to determine the estrous cycle. A sterile cotton swab was inserted into the rat’s vagina and rotated gently, rotated a few times, and removed. The mucus with vaginal epithelial cells was smeared on the slide in the same direction and stained with methylene blue. The changes in vaginal epithelial cells were observed under a light microscope, and the estrous cycle was assessed until the end of the experiment.

### Serum sex hormones and blood lipid indexes

After anesthesia, blood was collected through the abdominal aorta, centrifuged at 3000 rpm for 15 min, and stored at −80°C for later use. The serum T, E2, LH, FSH, SHBG, LCN-2, IL-1, IL-1β, IFN-γ, TNF-α, and IL-6 concentrations were determined by using an ELISA kit. Given that free testosterone and free androgen index (FAI) are the most sensitive indicators for determining hyperandrogenism, free T = total testosterone T/100 × (2.28−1.38 log SHBG/100), FAI = [T (pg/mL) ×100]/SHBG (pg/mL) (31). The concentrations of the blood lipid biochemical indicators TCHOL, TG, HDL-C, and LDL-C were measured by an automatic biochemical analyzer. The specific operation was carried out in strict accordance with the instructions. All the statistics are shown in **Table 2**.

**Table 2:** Body weight and serum indexes.

Characteristic	Control	Model	Met	High-dose	Z-dose	Low-dose
**Body weight (g)**	323.82±6.56	406.74±6.30***	395.49±6.32	378.57±7.33^##^	402.70±9.10	417.10±8.40
**LH (mIU/ml)**	3.95±0.36	4.35±0.28	4.22±0.36	4.48±0.33	4.75±0.35	4.96±0.40
**FSH (IU/L)**	3.60±0.19	1.36±0.05***	2.79±0.09**^###^**	2.31±0.13**^###^**	1.94±0.10**^##^**	1.81±0.10**^#^**
**LH/FSH**	1.12±0.12	3.21±0.19 ***	1.54±0.17**^###^**	1.99±0.16**^##^**	2.54±0.26	2.87±0.33
**Free androgen (Pg/ml)**	0.23±0.01	0.80±0.06	0.26±0.01	0.30±0.01	0.37±0.02	0.58±0.02
**SHBG (nmol/L)**	26.41±0.58	14.36±0.95***	27.08±0.82**^###^**	26.33±0.66**^###^**	20.94±0.57**^###^**	26.72±0.37**^###^**
**FAI**	37.85±1.68	247.10±21.06***	42.78±1.61**^###^**	50.71±1.86**^###^**	78.11±3.18**^###^**	104.44±6.33**^#^**
**E2 (Pmol/L)**	5.38±0.17	1.69±0.09***	4.68±0.17**^###^**	4.68±0.21**^###^**	3.87±0.17**^###^**	3.78±0.09**^###^**
**FBG (mmol/L)**	5.47±0.11	6.03±0.15*	5.91±0.17	5.29±0.12**^#^**	5.52±0.17**^#^**	5.72±0.21
**FINS (mIU/L)**	45.56±6.13	63.56±8.39**	55.49±12.89	53.32±8.33**^#^**	53.06±14.03**^#^**	51.45±11.95**^#^**
**HOMA-IR**	11.17±2.00	16.99±1.46***	14.76±3.90	12.15±2.07**^##^**	12.03±4.04^##^	12.44±2.96
**ISI**	-2.10±0.12	-2.36±0.18**	-2.20±0.23	-2.33±0.19	-2.26±0.20	-2.17±0.27**^#^**
**TCHOL (mmol/L)**	4.07±0.12	7.17±0.51**	4.21±0.24**^##^**	4.15±0.25**^##^**	5.00±0.25**^#^**	5.61±0.26
**TG (mmol/L)**	0.85±0.06	1.78±0.12***	0.97±0.04**^##^**	1.12±0.06**^##^**	1.20±0.05**^##^**	1.44±0.10
**LDL-C (mmol/L)**	0.98±0.16	3.79±0.27***	1.57±0.20**^###^**	1.32±0.17**^###^**	1.90±0.24**^###^**	2.54±0.23**^###^**
**HDL-C (mmol/L)**	2.89±0.09	1.06±0.13***	2.75±0.14**^###^**	2.78±0.11**^###^**	1.72±0.08**^###^**	2.44±0.15**^###^**

Remarks: “***”means there is a very significant difference between the model and the control group (*P* < 0.001), “**” means there is a significant difference between the model and the control group (*P* < 0.01),“*”means there is a difference between the model and the control group (*P* < 0.05); “###”means there is a very significant difference between the administration group and the model group (*P* < 0.001), “##”means there is a significant difference between the administration group and the model group (*P* < 0.01), “#”means there is a difference between the administration group and the model group (*P* < 0.05). All the above results, *n*=8~12.

### Blood glucose, insulin concentration, and oral glucose tolerance test

Blood was collected from the tail vein. The concentration of FBG was measured by using a Roche glucometer, and the concentration of FINS was measured by using an ELISA kit. The degree of IR was assessed by the IR index (HOMA-IR) and insulin sensitivity index (ISI). HOMA-IR=FBG (mmol/L) × FINS (mIU/L)/22.5. ISI = In [1/FINS (mIU/L) × FBG (mmol/L)] (32). The rats were fasted from food and water for 12 h, and the FBG of the rats was measured. The next morning, the rats were given 50% glucose solution by gavage at 3 g/kg. Blood was drawn through the tail vein at 0, 30, 60, 90, and 120 min to measure the blood glucose value. The area under the OGTT curve (AUC) was drawn, and the AUC results were calculated.

### Histological observation of rat ovary, adipose, and liver tissue

The bilateral ovaries and abdominal fat tissue were quickly removed on ice after collecting blood, weighed, photographed, and recorded. The left ovary was fixed in 4% paraformaldehyde solution, and the right ovary was frozen at −80°C for later use. At the same time, the fat around the ovary and livers was collected for pathological sections. Hematoxylin–eosin (HE) staining was used to observe the morphology of ovarian and adipose tissues around the ovary. Masson staining and Oil red O staining were used to observe the pathological conditions of the liver. With ovarian HE pathological section as an example, we removed the ovarian tissue fixed with 4% paraformaldehyde, routinely dehydrated the tissue, embedded it in paraffin, and cut the ovarian tissue into 5 μm-thick sections. The sections were then routinely deparaffinized, hydrated, stained with HE, and covered with neutral gum sheet. Finally, the pathological changes in the ovarian, liver, and adipose tissues of rats in each group were observed by using an upright optical microscope or digital pathological slice scanning system.

### Real-time quantitative PCR

Ovarian tissue was lysed, and the supernatant was obtained. A Tissue RNA Purification Kit PLUS Kit was used to extract 25 μL of RNA from the spin column for RNA extraction. A microspectrophotometer (Thermo, NANODROP 2000) was used to measure the RNA concentration. cDNA was prepared by reverse transcription using Color Reverse Transcription Kit. qRT-PCR reactions were prepared with 2× Color SYBR Green qPCR Master Mix and detected with the ABI7500 system (Applied Biosystems, Thermo Fisher Science, Inc., Waltham, MA, USA). The amplification conditions were as follows: 95°C for 5 min, 95°C for 15 s, and 60°C for 30 s for a total of 40 cycles. The specific primers used are shown in **Table 1**, and the number of each target gene was normalized to *Gapdh* (internal reference gene).

### Western blot

About 30 mg of ovarian tissue from every rat was prepared on ice. The total protein from ovarian tissue was extracted with RIPA lysis buffer (CWBIO) and protease and phosphatase inhibitors. After the protein concentration was measured by BCA, the proteins were separated by a 10% SDS-PAGE electrophoresis system and transferred to PVDF membranes. The membranes were blocked with 5% skim milk at room temperature for 3 h, incubated with the primary antibody overnight at 4°C, incubated with the secondary antibody of the same source for 1 h, developed with ECL reagent (Billerica, USA), and imaged with a Tanon 5200 chemiluminescence system (Shanghai Tanon Technology, China). NF-κB p65(D14E12)XP(R)Rabbit mAb (Lot:13, Cell Signaling Technology, Inc.), phospho-NF-κB p65 (S536)(93H1)Rabbit mAb (Lot:17, Cell Signaling Technology, Inc.), NGAL (H-7) SAMPLE sc-515876(Lot#H1321, Santa Cruz Biotechnology), anti-β-actin Mouse Monoclonal Antibody (Lot:01264/00621, Beijing Kangwei Century Biotechnology Co., Ltd.), goat Anti-Rabbit IgG HRP Conjugated (Lot: 01334/10921, Beijing Kangwei Century Biotechnology Co., Ltd.), and goat Anti-Mouse IgG HRP Conjugated (Lot: 01325/30503, Beijing Kangwei Century Biotechnology Co., Ltd.) were utilized in this study.

### Statistics

The experimental data were processed by SPSS Statistics 26 software, and GraphPad Prism 8 was used for image processing. The measurement data conforming to the normal distribution were expressed as mean ± standard error (mean ± SEM), and the comparison between multiple groups was performed by one-way ANOVA. The LSD test was used for comparison between groups for homogeneity of variance, and the Dunnett’s T3 test was used for unequal variance. P<0.05 was considered to be statistically significant.

## Results

### MCDD slowed down the weight gain of PCOS-IR rats and significantly improved the abdominal fat weight of PCOS-IR rats

The PCOS-IR rat model was established by gavage of letrozole combined with high-fat diet, in which the obesity model induced by high-fat diet led to the IR phenotype. Considering the difference in energy between the high-fat diet and the control diet, the daily average food intake of each group was recorded while the food was free. The dynamic changes in food intake and food energy were used to help observe weight gain. We observed the change trends of body weight of rats in each group under the effect of drug intervention for six consecutive weeks, and the difference in body weight of each group was determined in the sixth week.

**Figure 2:**
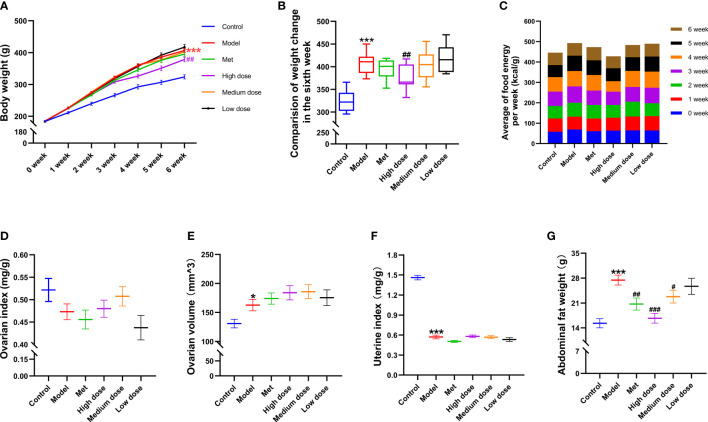
Effects of MCDD on body weight and organ index of rats. **(A)** The body weight change curve of the rats in each group for six consecutive weeks. **(B)** The body weight difference diagram of the rats in the six groups on the sixth week. **(C)** The average free food intake energy of the six groups of rats was counted every day. After sacrifice, the weight of ovary, uterus, and abdominal fat were weighed, and the ratio to body weight was **(D)** ovarian index. **(E)** ovarian volume (mm^3^) = (π/6) × long diameter (mm) × short diameter ^2^ (mm^2^). **(F)** uterine index. **(G)** abdominal fat index of rats. The above results are expressed as mean ± SEM, *n*= 8~12. ***P < 0.001, *P < 0.05, vs. control group, ^###^ *P* < 0.001, ^##^ *P* < 0.01, ^#^ *P* < 0.05, vs. model group.

**Figures 2A–C** show that the energy intake of the rats in each group was equal every week (**Figure 2C**), and there was no difference in the body weight of the rats in the zero week. In the sixth week, the model group showed a significant increase in body weight compared with the control group (***P<0.001; **Figures 2A, B**). Compared with the model group, the metformin group showed a tendency to reduce body weight, whereas the high-dose group significantly decreased the body weight of rats (^##^ *P*<0.01). No difference in weight loss was found between the medium-dose and low-dose groups (**Figure 2A**). The results suggested that a high-fat diet could effectively establish an obesity model, and the addition and subtraction of MCDD reduced body weight in a dose-dependent manner.

To observe the general condition of the organ index of the rats in each group after modeling and administration, the ovary, uterus, and abdominal fat were weighed and counted immediately after the rats were sacrificed. As shown in **Figures 2E, F**, the ovarian index results (**Figure 2D**) revealed that the wet weight of the ovaries of the rats in the model group decreased compared with that in the control group. There was an upward trend in the groups, but no difference was noted. The ovarian volume (**Figure 2E**) showed a difference between the model group and the control group (*P<0.05), and the volume had a tendency to increase. In terms of shape and volume, the ovaries in the control group were light red and oval, with nodular follicles on the surface. The ovaries in the model group were dark red, and the follicles increased in volume and showed white grape-like aggregates. The ovaries in each drug group were mostly pale pink, with occasional white vesicular accumulations. The results of the uterine index (**Figure 2F**) showed that the uterine index of the model group decreased compared with that of the control group (***P<0.001). The natural state of rat uterus was slender, and the uterine aperture was about 1/2 narrower than that of the control group. The results of abdominal fat wet weight (**Figure 2G**) showed that the abdominal fat weight of the model group increased compared with that of the control group (***P<0.001). Compared with the model group, the abdominal fat weight of the four administration groups decreased in a dose-dependent manner; the highest reduction was observed in the high-dose group (^###^ *P*<0.001). The above results suggested that letrozole combined with a high-fat diet could reduce the uterine and ovarian indices, increase the ovarian volume, and significantly increase the weight of abdominal fat in PCOS-IR rats. MCDD could reduce abdominal fat in a dose-dependent manner.

### MCDD improves PCOS-IR ovarian function

**Figure 3:**
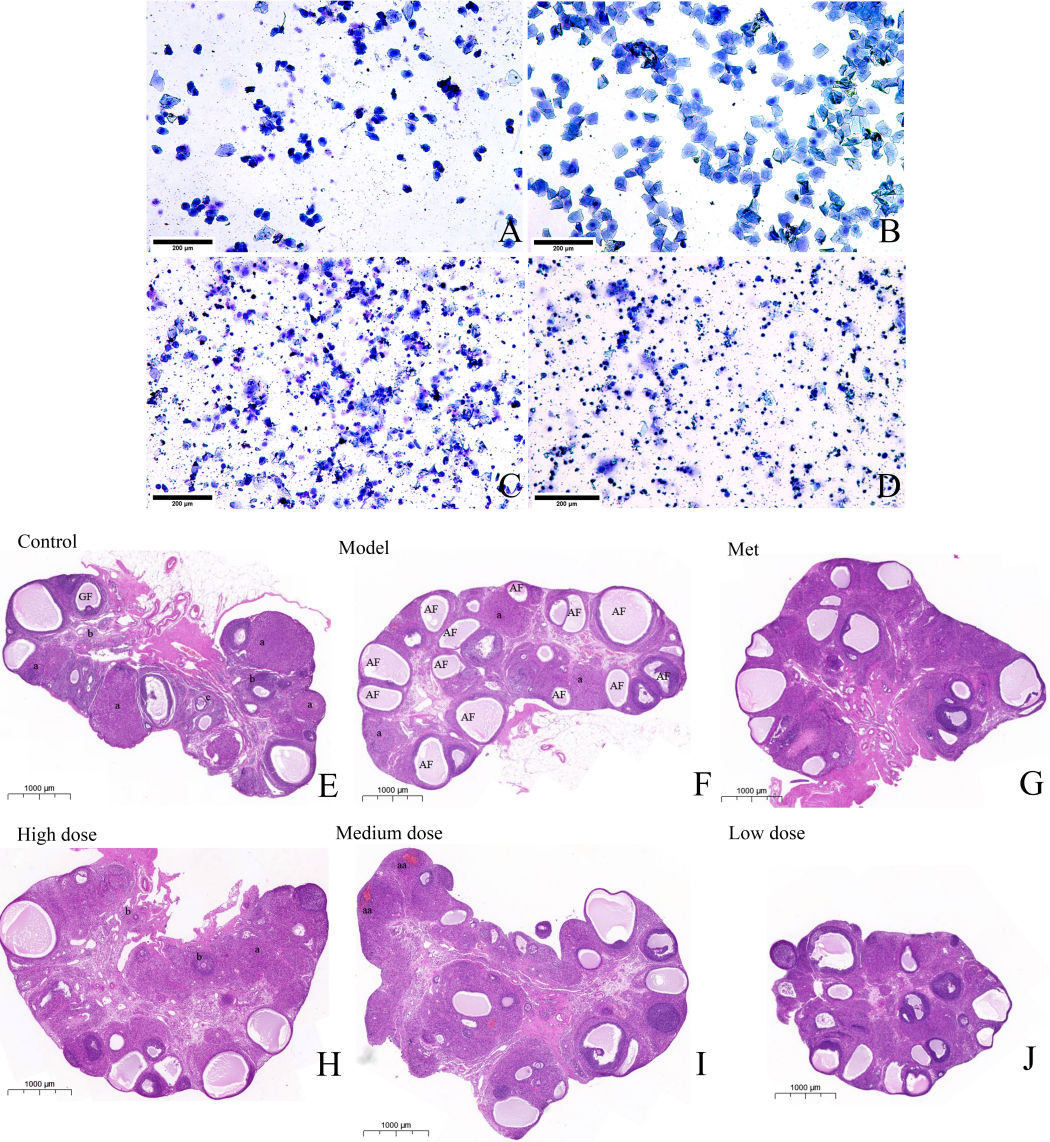
MCDD regulates PCOS-IR estrous cycle disorder and improves ovarian pathological characteristics. The vaginal smear method was used to observe the estrous cycle stages of the rats in each group under an upright microscope after methylene blue staining **(A)** Proestrus, **(B)** Estrus, **(C)** Metestrus, **(D)** Diestrus. The above results, *n*=12 (scan bar:200 μm). After the rats were sacrificed, the left ovaries were collected, soaked in 4% paraformaldehyde, representative images of H&E-stained rat ovaries for the indicated groups. **(E)** H&E-stained rat ovaries of control group (scan bar:1000 μm). GF (Gratian follicle), “a” means corpus luteum,”b” means Primordial follicle (PmF), “c” means Primary follicle (PrF). **(F)** H&E-stained rat ovaries of model group. AF (Atretic follicle). **(G)** H&E-stained rat ovaries of Met group. **(H)** H&E-stained rat ovaries of high dose group. **(I)** H&E-stained rat ovaries of medium dose group. “aa” means fresh corpus luteum (there is blood remaining in the corpus luteum, which is a relatively fresh corpus luteum after ovulation). **(J)** H&E-stained rat ovaries of low dose group. The above results, *n* = 3.

Two weeks before the end of the experiment, the estrous cycle stages of the rats in each group were continuously observed at ten o’clock in the morning to determine the disorder of the estrous cycle of the rats in the model group and the recovery of the estrous cycle of the rats in the administration group. The vaginal smear method was used to indirectly reflect ovarian ovulation features. As shown in **Figures 3A–D**, (A) the characteristics of rat in the proestrus: irregularly shaped nucleated epithelial cells were distributed in clusters and concentrated in sheets, and the nuclei were clearly visible, (B) the estrus stage of rat: a large number of sheet-like anucleated keratinocytes were evenly distributed, (C) the metestrus: nucleated epithelial cells, keratinocytes, and leukocytes were evenly distributed in proportion, (D) the diestrus: a large number of leukocytes were concentrated in black spheres under low magnification, and nuclei could be observed under the high-magnification visual field, and another part of the white blood cells was distributed in a dissipated state. Observation and recording for 14 consecutive days showed that the rats in the control group could maintain a relatively regular estrous cycle, and more keratinocytes appeared in estrus, whereas the model group had more keratinocytes. It continued to stay in the diestrus with a large number of leukocytes, which showed the disorder of the estrous cycle, thereby indicating that the letrozole model was successful. Meanwhile, metformin and MCDD improved ovarian function. HE staining was conducted to observe the pathological changes of the ovary. As shown in **Figures 3E–J**, the whole ovary was observed by scanning electron microscopy. Primordial follicles at different developmental stages were found in the control group. The follicles at all levels of growth were clearly visible, the follicle granulosa cells were thick, and there were few atretic follicles. The number of the model group’s atretic follicles significantly increased, the granular layer of different sizes was thin and diffusely distributed, no follicles at all stages were seen, and the number of corpora lutea was significantly reduced, indicating anovulation. The cystic dilated atretic follicles in the metformin group decreased, and the number of corpora lutea gradually increased. The high-dose, medium-dose, and low-dose groups exhibited different degrees of improvement. Cystic atresia follicles were significantly reduced, fresh corpus luteum and follicles at all levels were observed in the ovarian cortex, and granulosa cells were thick and arranged completely.

### MCDD relieves the fatty liver phenomenon and the size of adipocytes

**Figure 4:**
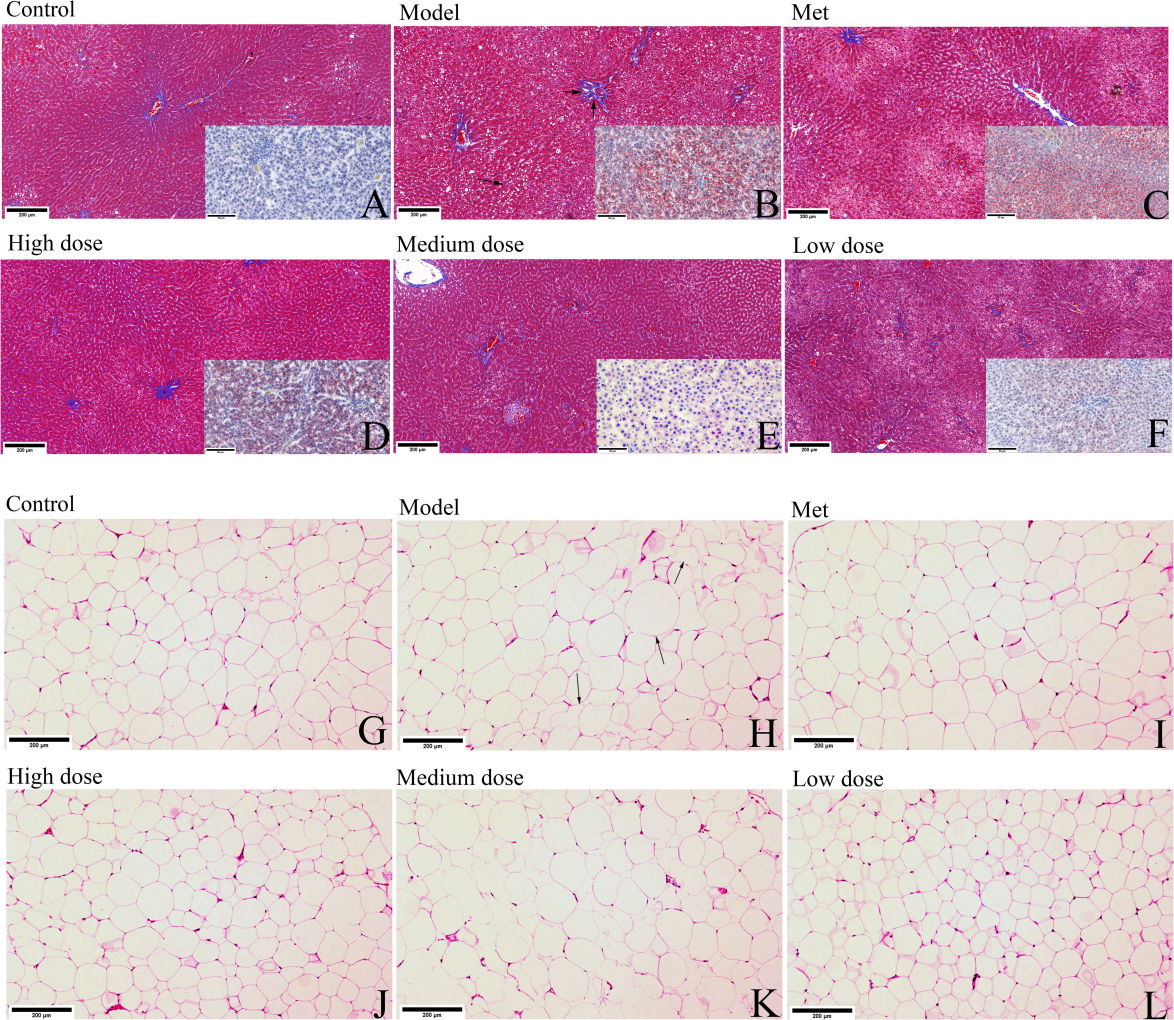
MCDD alleviates the phenomenon of PCOS-IR fatty liver and adipocyte enlargement. After the rats were sacrificed, the livers and small pieces of adipose tissue around ovary were collected, soaked in 4% paraformaldehyde, and stained separately with Masson trichrome and oil red O staining or hematoxylin-eosin. **(A–F)** The Masson trichrome-stained sections of the rat liver tissue in each group are shown in the large picture (scan bar:200 μm), and the oil red O-stained sections are shown in the small picture in the lower right corner (scan bar:50 μm). (Arrows in Masson trichrome staining show vacuolar fat vesicles, lipid deposits in hepatocytes in Oil Red O-stained sections are stained red). **(G–L)** HE sections of adipose tissue of rats in each group (scan bar:200 μm). (In the model group, arrows indicate fat cells with increased volume and irregular shapes). The above results, *n*=3.

HE, Masson trichrome, or Oil red O staining was used to observe the pathological changes of the adipocytes and liver tissues in the six groups of rats. As shown in **Figures 4A–L**, the Masson slices of the liver were first observed. The hepatic lobules of the control group were radially arranged from the central vein and surrounding stem cells in a plate-like distribution, and the hepatocytes were large in size and rich in the cytoplasm (**Figure 4A**). In the model group, different sizes of round, tension vacuoles in the cytoplasm of hepatocytes (shown by an arrow) were located in the periphery of the hepatic lobule, showing vesicular steatosis and focal accumulation of inflammatory cells and hepatic granuloma in the hepatic portal area (shown by two arrows) (**Figure 4B**). In the metformin group, the infiltration of inflammatory cells between the hepatocyte cords was alleviated, and the distribution of vesicular vacuolar fatty degeneration was reduced. The fatty liver of rats in the high-, medium-, and low-dose groups gradually disappeared, and the hepatic lobules were arranged radially and neatly around the central vein. The liver oil red O slices revealed that the liver lobules in the control group were arranged radially and neatly, and the whole area was not red. The model group showed large vacuoles, which were stained red due to the lipid deposition of liver cells, and the number of vacuoles was reduced. Observing the HE-stained sections of adipose tissue, the adipocytes of the rats in the control group were neatly arranged in an ellipsoid shape and evenly distributed in size, the adipocytes in the model group increased significantly in size, and some were irregularly arranged in shape (shown by arrows). The adipocytes in each administration group recovered to ellipsoid shape, and most of them were evenly distributed (**Figures 4G–L**).

The above pathological results suggested that metformin and MCDD could improve the pathological state of ovarian cystic dilatation, increase the number of corpus luteum, and improve the phenomenon of fatty liver and abnormal enlargement of adipocytes.

### MCDD regulates PCOS-IR by improving hormone levels

**Figure 5:**
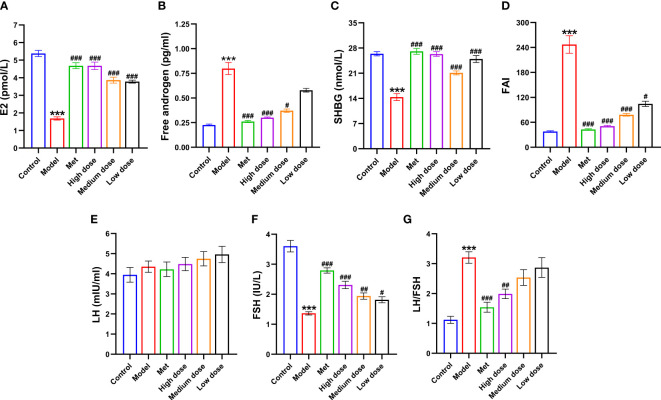
Effects of MCDD on serum factors in PCOS-IR rats. **(A)** Estradiol, **(B)** Free androgen, **(C)** Sex hormone binding globulin, **(D)** Free androgen index, **(E)** LH, **(F)** FSH, **(G)** LH/FSH. The serum sex hormones and other indicators of rats were measured by enzyme-linked immunosorbent assay kit. The above results are expressed as mean ± SEM, *n* = 10~11. ***P < 0.001, vs. control group, ^###^ *P* < 0.001, ^##^ *P* < 0.01, ^#^ *P* < 0.05, vs. model group.

To further observe the changes in estradiol (E2), free androgen, SHBG, and FAI in each group of rats, as well as to explore the model formation in the PCOS-IR model group with the efficacy of MCDD, the content of the first three sex hormones in the serum of rats was detected by ELISA. The FAI was calculated, and the results are shown in **Figures 5A–D**. Compared with the control group, the E2 and SHBG contents in the model group decreased (***P<0.001), and the free androgen and FAI increased (***P<0.001). Compared with the model group, the E2 content of the metformin group and the high-dose, medium-dose, and low-dose groups increased significantly (^###^ *P*<0.001), but the high-dose group had the most obvious effect. The content of free androgen in the metformin and high-dose groups decreased significantly (^###^ *P*<0.001), whereas that of the medium-dose group decreased (^#^ *P*<0.05). There was no significant difference, but a decreased trend was observed in the low-dose groups, and the effect was more obvious in the high-dose and medium-dose groups. SHBG among the four administration groups increased (^###^ *P*<0.001), and the medium-dose group showed the best effect, suggesting that MCDD could significantly reduce androgen in rats, which greatly increased the number of SHBG bound to it. The FAI significantly decreased in the metformin, high-dose, medium-dose (^###^ *P*<0.001), and low-dose groups (^#^ *P <*0.001), and the effect was better in the high- and medium-dose groups. The above results suggested that MCDD could increase E2 levels in a dose-dependent manner, reduce serum free androgen and SHBG content, and improve the androgen index. To observe the changes in luteinizing hormone (LH) and follicle-stimulating hormone (FSH) in each group and determine the efficacy of MCDD, the serum LH and FSH contents of rats were detected by ELISA. As shown in **Figures 5E–G**, the FSH content of rats in the model group was the lowest compared with that in the control group (***P<0.001), whereas the LH content increased and LH/FSH significantly increased (***P <0.001), indicating successful modeling. Compared with the model group, the four groups of administration groups all exhibited an increase in the FSH content, among which the model group had extremely significant differences with the metformin and high-dose groups (^###^ *P*<0.001) and significant differences with the medium-dose (^##^ *P*<0.01) and low-dose groups (^#^ *P*<0.05). Compared with the model group, the metformin group showed a downward trend in LH content. For LH/FSH, both the metformin and high-dose groups showed a decrease, and there was a very significant difference with the metformin (^###^ *P*<0.001) and high-dose (^##^ *P*<0.01) groups. The above results suggested that letrozole combined with a high-fat diet could increase the ratio of LH/FSH in rats, and MCDD inhibited the increase in the LH/FSH ratio in a dose-dependent manner and increased the content of FSH in the body.

### MCDD improved IR by decreasing the area under the glucose tolerance curve, FBG, and FINS, thereby slowing down HOMA-IR, increasing ISI, and significantly changing four biochemical indicators in PCOS-IR rats

**Figure 6:**
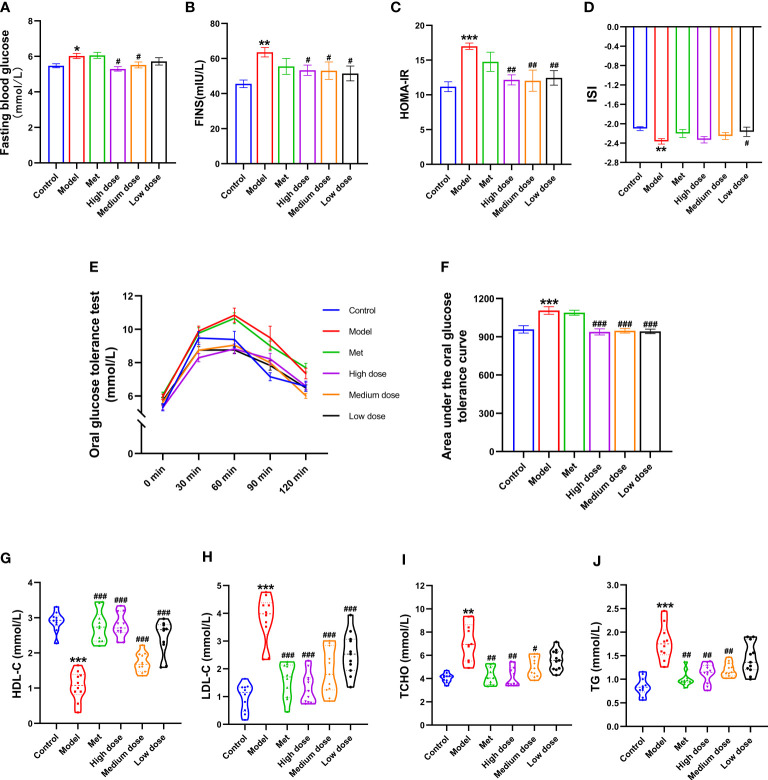
Effects of MCDD on insulin resistance in PCOS-IR rats. **(A)** Fasting blood glucose of rats was measured by glucose oxidase method after fasting for 12 hours, **(B)** FINS, **(C)** HOMA-IR, **(D)** ISI, **(E, F)** OGTT area under the curve, the changes of serum IR-related indexes of rats were detected by enzyme-linked immunosorbent assay kit. **(G–J)** four blood lipid biochemical indicator were measured by automatic biochemical analyzer. The above results are expressed as mean ± SEM, *n* = 8~12. ***P < 0.001, **P < 0.01, *P < 0.05, vs. control group, ^###^ *P* < 0.001, ^##^ *P* < 0.01, ^#^ *P* < 0.05, vs. model group.

To explore the IR index in the PCOS model and the efficacy of MCDD on IR, glucose oxidase was used to collect blood from the tail vein of rats to detect the FBG. OGTT was detected at five time points of 0, 30, 60, 90, and 120 min, and the area under the OGTT curve of each group of rats was calculated to observe its changes. The results are shown in **Figures 6A, B, E, F**. Compared with the control group, the FBG of the model group increased (*P <0.05). The FBG of the high- (^#^ *P <*0.05), medium- (^#^ *P <*0.05), and low-dose groups decreased compared with that of the model group. The AUC of the model group was the highest, and the AUCs of the four administration groups were all lower than that of the model group. After calculation of the AUC, the model group showed a very significant difference compared with the control group (***P<0.001). Compared with the model group, the high-, medium-, and low-dose groups were comparable, with extremely significant differences (^###^ *P <*0.001). The results suggested that MCDD could reduce FBG in PCOS-IR rats and improve abnormal oral glucose tolerance. To verify the IR of rats in the model group and the efficacy of MCDD on IR, ELISA kits were used to detect FBG, FINS, HOMA-IR, and ISI. The IR index and sensitivity index were calculated. The results are shown in **Figures 6C, D**. Compared with the control group, the HOMA-IR of the model group increased (***P<0.001), and the ISI significantly decreased (**P<0.01). Compared with the model group, the HOMA-IR in the administration group decreased (^##^ *P*<0.01). Therefore, the PCOS-IR model was stable, and MCDD increased insulin sensitivity and relieved IR in a dose-dependent manner on PCOS-IR. To further verify the changes in blood lipid and biochemical indicators *in vivo* after IR was induced by the obesity model and determine the therapeutic effect of MCDD on PCOS-IR, ELISA kits were used to detect four serum lipid biochemical indicators in rats and assess obesity-related indicators other than body weight in rats. As shown in **Figures 6G–J**, compared with the control group, HDL-C in the model group decreased (***P <0.001), LDL-C increased (***P <0.001), and TCHO and TG increased (***P <0.001). Compared with the model group, the HDL-C concentrations of the four administration groups increased (^###^ *P*<0.001), and the medium-dose group increased most significantly. The LDL-C levels of the four administration groups decreased (^###^ *P <*0.001), and the high-dose group had the most significant decrease. In terms of the TCHO index, the metformin (^##^ *P*<0.01), high-dose (^##^ *P*<0.01), and medium-dose groups (^#^ *P*< 0.05) decreased compared with the model group. For the TG index, compared with the model group, the four administration groups all decreased; the metformin, high-dose, and medium-dose groups were all significantly different from the model group (^##^ *P <*0.01). The above results suggested that a high-fat diet could lead to obesity in rats and make the four blood lipid indexes abnormal. MCDD could improve the abnormal blood lipid index in a dose-dependent manner.

### MCDD can reduce the levels of serum inflammatory factors IL-1, IL-6, IL-1β, TNF-α, and IFN-γ

**Figure 7:**
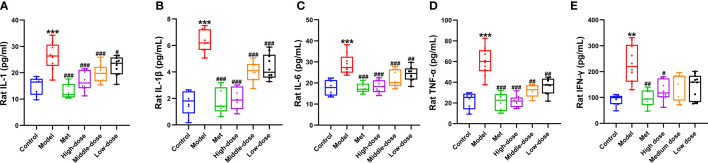
Effect of MCDD on serum inflammatory factors **(A)** IL-1, **(B)** IL-1 β, **(C)** IL-6, **(D)** TNF-α, **(E)** IFN-γ. The enzyme-linked immunosorbent assay kit was used to detect the changes of serum NF-κB pathway-related inflammatory factors in rats. The above results are expressed as mean ± SEM, *n* = 8~11. ***P < 0.001, **P < 0.01, vs. control group, *^###^P* < 0.001, ^##^ *P* < 0.01, ^#^ *P* < 0.05, vs. model group.

To explore the relationship between PCOS-IR and inflammatory environment-related indicators, ELISA was used to measure the changes in IL-1, IL-6, IL-1β, TNF-α, and IFN-γ in serum of rats. The results are shown in **Figures 7A–E**. Compared with the control group, the five inflammatory indexes increased in the model group, among which IL-1, IL-6, IL-1β, and TNF-α were significantly different (***P <0.001). A significant difference was observed in IFN-γ (**P<0.01). Compared with the model group, the five inflammatory indexes all showed a decreasing trend. Among them, the four inflammatory indexes of IL-1, IL-6, IL-1β, and TNF-α extremely decreased in the four administration groups (*^###^P*<0.001). Compared with the model group, extremely significant differences were found in IL-1β (*^###^P*<0.001), IL-6 and TNF-α (*^##^P*<0.01), and IL-1 levels (*^#^P*<0.01). For the IFN-γ index, significant differences were observed between the metformin and model groups (*^##^P <*0.01) and between the high-dose and model groups (*^#^P <*0.05). The above results showed that the levels of inflammatory index factors IL-1, IL-6, IL-1 β, TNF-α, and IFN-γ in the PCOS-IR model significantly increased, whereas MCDD could reduce IL-1, IL-6, IL-1β, TNF-α, and IFN-γ in a dose-dependent manner. The contents of IL-6, IL-1β, and TNF-α suggested that PCOS-IR was related to the inflammatory microenvironment. MCDD may exert its therapeutic effect by mediating the PCOS-IR inflammatory pathway.

### MCDD may improve the symptoms of IR in PCOS by mediating the NF-κB/LCN-2 signaling pathway and regulating the molecules of the insulin signaling pathway

**Figure 8:**
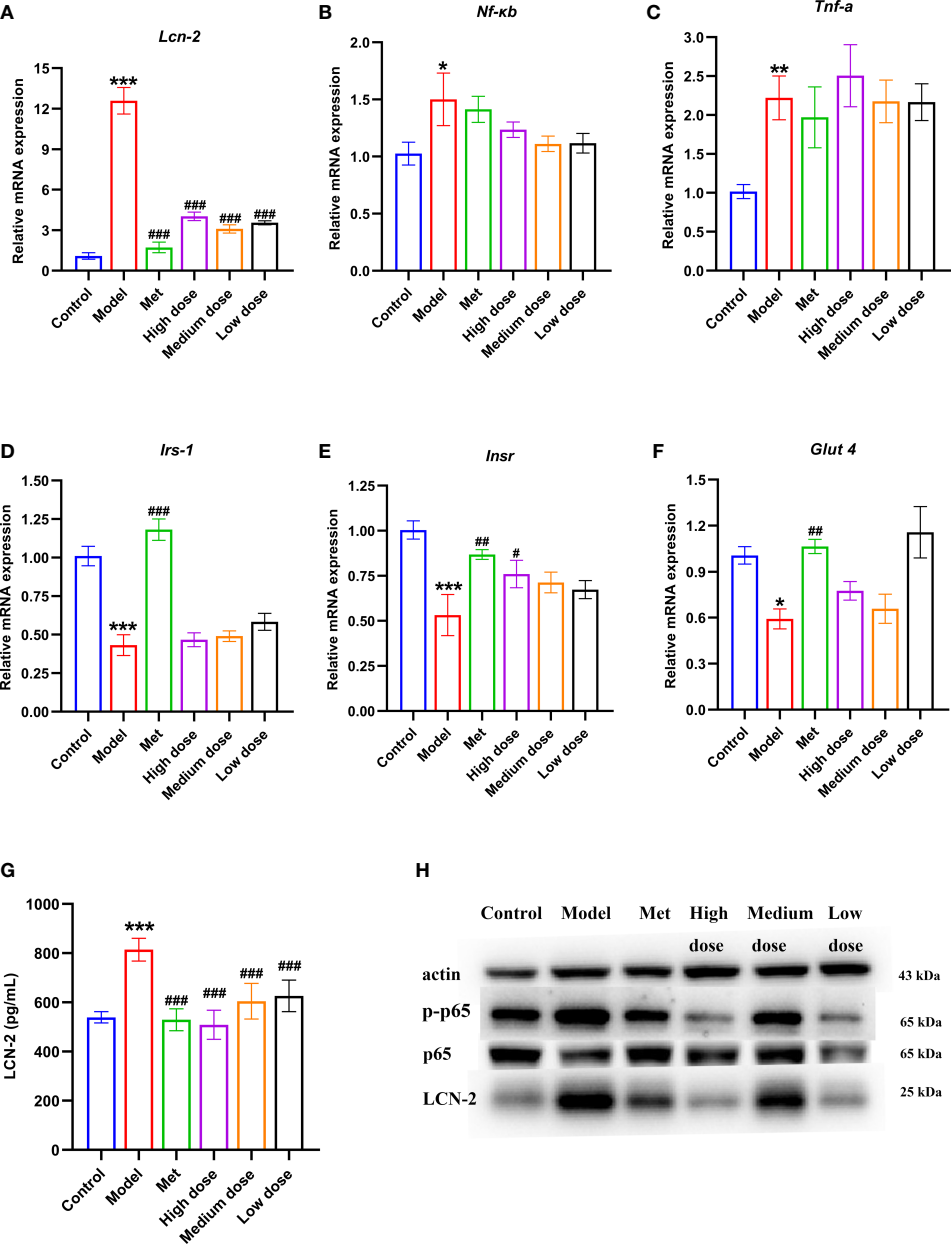
Effects of MCDD on serum LCN-2 content, genes of insulin signal transduction pathway, inflammatory factors of NF-κB inflammatory pathway, LCN-2 and NF-kB p-p65 protein in ovarian tissue. qRT-PCR detection of gene expression of *IRS-1*, *INSR* and *GLUT 4* in rat ovarian tissue **(A)** *LCN-2*, **(B)** *NF-κB* **(C)** *TNF-α*, **(D–F)** insulin signal transduction pathway, *n*=5. **(G)** Changes of serum LCN-2 levels in rats detected by enzyme-linked immunosorbent assay kit, *n*=8~12. **(H)** Western blot detection of p-p65, p65 and LCN-2 protein expression in NF-κB inflammatory pathway in rat ovarian tissue, *n*=3, the above results are expressed as mean ± SEM. ***P < 0.001, **P < 0.01, *P < 0.05 vs. control group, ^###^ *P* < 0.001, ^##^ *P* < 0.01, ^#^ *P* <0.05, vs. model group.

To verify that LCN-2 may be a potential target molecule related to IR in PCOS-IR, ELISA was used to observe the changes in LCN-2 in serum of rats. The results are shown in **Figure 8G**. The serum content increased in the model group compared with that in the control group (***P<0.001). Compared with the model group, the four administration groups presented extremely significant differences (^###^ *P <*0.001). All showed a downward trend, but the decrease was most obvious in the high-dose group. The above results showed that serum LCN-2 levels were abnormally increased in PCOS-IR rats. Metformin and different doses of MCDD could inhibit the increase in LCN-2, suggesting that the effect of MCDD may be related to the potential target of IR, namely, the molecule LCN-2.

To verify that MCDD treats PCOS-IR by regulating insulin signaling pathway factors, qRT-PCR was used to detect the gene expression levels of insulin signaling pathway-related *Insr*, *Irs-1*, and *Glut4* in rat ovarian tissue. As shown in **Figures 8D–F**, the expression levels of *Irs-1*, *Insr*, and *Glut4* in the model group significantly decreased compared with those in the control group. Compared with the model group, the expression levels of *Irs-1*, *Insr*, and *Glut4* all increased in the administration groups. For the *Irs-1* gene, a very significant difference was found between the model group and the control group (***P<0.001), and a very significant difference was noted between the metformin group and the model group (^###^ *P <*0.001). For the *Insr* gene, the model group was significantly different from the control group (***P<0.001), the metformin group was significantly different from the model group (^##^ *P*<0.01), and the high-dose group was different from the model group (^#^ *P*<0.05). For the *Glut4* gene, a difference was observed between the model group and the control group (*P<0.05), whereas a significant difference was noted between the metformin group and the model group (^##^ *P <*0.01). The above results showed that the downstream signaling molecules of the insulin signaling pathway in the ovary of PCOS-IR rats were inhibited. MCDD may restore the expression levels of *Insr*, *Irs-1*, and *Glut4*; strengthen insulin signaling; and exert its efficacy. To further verify that MCDD could improve the inflammatory microenvironment and regulate LCN-2 to exert its efficacy, qRT-PCR was performed to detect the levels of inflammatory factors *Lcn-2*, *Nf-κb*, and *Tnf-α* in rat ovarian tissue. The results of gene expression are shown in **Figures 8A–C**. Compared with the control group, the expression folds of *Lcn-2* (***P<0.001), *Nf-κb* (*P <0.05), and *Tnf-α* (**P <0.01) were significantly upregulated in the model group, especially *Lcn-2*. Thus, LCN-2 may be closely related to the pathogenesis of PCOS-IR. Compared with the model group, *Lcn-2* expression was inhibited and decreased in each administration group, suggesting that metformin and MCDD could improve the inflammatory microenvironment. The previous results in **Figure 7** showed that PCOS-IR was associated with inflammatory factors in an inflammatory environment. MCDD could reduce the content of inflammatory factors to varying degrees. To further verify the changes in NF-κB/LCN-2 pathway proteins in the ovary, Western blot was used to detect the expression levels of p-p65, p65, and LCN-2 in ovarian tissues of six groups of rats. The results are shown in **Figure 8H**. Compared with the control group, the expression of p-p65 and LCN-2 in the model group increased, whereas the expression of p65 did not change remarkably. Compared with the model group, the expression of p-p65 protein in the four administration groups decreased. The high-dose group had the most significant decrease, whereas the metformin and high-dose groups had the most significant decrease in the expression of LCN-2 protein. The above results suggested that NF-kB p-p65 and LCN-2 protein increased in ovarian tissues of the PCOS-IR rat model. Thus, metformin and MCDD may downregulate NF-κB pathway inflammatory factors and LCN-2 to exert their efficacy.

## Discussion

PCOS-IR is one of the most prevalent gynecological diseases in adolescent- and reproductive-age women, it brings a great burden on women’s psychological and physical health (33–36). HI, dysregulation of the hypothalamus-pituitary-ovary axis, abnormal adrenal androgen secretion, familial genes, and inflammation may affect the occurrence and development of PCOS-IR to varying degrees, but the mechanism remains unclear. Therefore, finding a TCM compound with remarkable curative effect and minimal side effects is of great significance. PCOS is closely associated with obesity, in which weight management is a first-line treatment (37), so maintaining a healthy weight is key in the management of PCOS-IR. We used MCDD as the research object to explore its pathway transduction mechanism and possible effector targets in improving PCOS-IR. Previous studies have found that MCDD has a significant effect on PCOS-IR in clinically obese patients, especially in terms of weight loss. Moreover, it can play a corresponding effect in animal experiments (23, 26–30). Our results showed that different doses of MCDD could improve the body weight and wet weight of abdominal fat and restore the ovarian function of rats in a dose-dependent manner. In addition to the pathological changes of the ovary, we observed remission of liver and adipose tissue in the model group after MCDD intervention. MCDD may alleviate fatty liver. Notably, we found that MCDD may play a therapeutic effect by downregulating the molecular expression of LCN-2/NF-κB p-p65 and upregulating the gene expression of the insulin signaling pathway, suggesting a relationship between LCN-2 and the NF-κB signaling and insulin signal transduction pathways.

MCDD could significantly exert a pharmacodynamic effect on PCOS-IR rats. First, our results showed that MCDD could significantly improve abdominal obesity, which was consistent with the clinical treatment of obese patients with PCOS (23, 28, 29). The results of ovarian and uterine indexes did not change significantly, and they were consistent with our previous experimental results. We found that Xu Haiyan and others believe that different modeling cycles will affect the change in uterine index, and its measurement is easy to be deviated by the standardization of operators (38, 39). Different doses of MCDD can restore the ovarian function of PCOS-IR rats, improve the disorder of the estrous cycle, restore fatty liver, reduce tissue inflammation accumulation, and improve the shape of adipocytes. The pathophysiology of non-alcoholic fatty liver disease (NAFLD) in women with PCOS is associated with common metabolic disorders of the syndrome, such as IR, obesity, and dyslipidemia. Studies have shown that excess androgen may increase the risk of developing NAFLD in these patients. After statistical adjustment for body mass index (BMI) and IR, a case–control study showed that women with PCOS and hyperandrogenemia are more likely to develop NAFLD than non-hyperandrogenic PCOS and healthy women (40). Significant morphological changes of fatty liver were observed in liver tissues, and these changes were associated with HOMA-IR (41–47). Women with PCOS usually show significant metabolic complications (3). Thus, observing liver and adipocytes pathological sections could help to judge whether the IR phenotype is successful or not. Different doses of MCDD obviously improved serum sex hormones, IR-related indexes (such as OGTT, HOMA-IR, and FINS), and blood lipid biochemical indexes. The efficacy of the high-dose MCDD group was better than that of the middle- or low-dose groups, and most of them tended to decrease the parameters of each index. The high-dose group in SHBG and HDL-C showed an upward trend, which was determined by the different index properties of SHBG and HDL-C. SHBG is a homodimer plasma glycoprotein that binds to sex hormones and regulates the biological activity of sex hormones, including testosterone, dihydrotestosterone (DHT) with high affinity, and estradiol with low affinity (48, 49). About 66% of testosterone in human serum binds to SHBG, 31% to albumin, and 1.5% to corticosteroid transporters; only 2% are free, and only free androgens are biologically active (50). Thus, SHBG is an important factor in regulating the concentration of serum free testosterone. After MCDD treatment, free androgen decreased while SHBG increased in the model rats. Besides, MCDD had little effect on LH due to the following reasons: LH is a hormone that is involved in the steroidogenesis of ovarian membrane cells. It depends on the regulation of the rate-limiting enzyme cytochrome P450c17 in ovarian membrane cells. It is also responsible for the increased androgen biosynthesis associated with PCOS (3). The changes in LH levels are affected by several factors: hyperandrogenemia enhances the hypothalamic gonadotropin-releasing hormone (GnRH) pulse frequency through the negative feedback of inhibitory steroids on LH secretion, which eventually leads to the increase in LH and androgen levels. Other factors such as anti-Mullerian hormone (AMH) and insulin may contribute to neuroendocrine dysregulation, which may contribute to enhanced androgen production in the ovary and be involved in the pathogenesis of this disease. Increased AMH levels in PCOS may promote GnRH release from the hypothalamus and lead to HA. Insulin may increase the frequency and amplitude of GnRH and LH pulse secretion by upregulating GnRH gene expression in hypothalamic neurons, which is mediated by activation of the MAPK pathway (3). In summary, in addition to GnRH regulation, LH may be affected and regulated by other pathways, such as AMH or insulin. The process is diverse and complex, and many factors should be considered. Therefore, we should pay attention to the more objective ratio of LH to FSH. In addition, the non-significant LH level in MCDD alleviated rats was different from clinical trials may be related to species differences. Similarly, MCDD can significantly increase HDL-C levels, and clinical studies have found that obese patients are at increased risk of cardiovascular disease and need to be treated for dyslipidemia; weight loss decreases serum triglyceride and LDL-C levels and increases HDL-C levels (51–54).

Reviewing the results of the positive drug metformin, metformin was found to have a significant effect in reducing LH/FSH and blood lipid biochemical indexes, compared with thiazolidinediones (THIAZOLES), insulin sensitizers are limited to metformin, which has different benefits for patients with PCOS, including improved weight management and glucose tolerance, reduced androgen production, and improved menstrual cycle and fertility (55). Insulin-sensitizing drugs was clinically used in PCOS-IR treatment because of the interaction between IR and PCOS. However, metformin had not significant effect on PCOS-IR rats in our study. The dose of our positive drug metformin was 500 mg bid (= 1000 mg/day) (56), this was also in line with the dose for clinical use in adults. However, the metformin product used this time is a coating tablet, in addition to metformin hydrochloride as the main component, other contents account for a part of the volume, which makes the content of metformin relatively reduced, so maybe the efficacy seems insufficient. Second, evidence-based guidelines recommend lifestyle changes as a first-line treatment for PCOS-IR. It has been reported that a healthy lifestyle such as exercise combined with metformin is more likely to improve obesity than metformin alone (37), possibly because we did not use exercise intervention, so the effect of metformin alone is not obvious. Finally, metformin, as an insulin sensitizer and hypoglycemic agent, regulates metabolism by inhibiting hepatic glycogen output, increasing glucose utilization in non-insulin-dependent tissues, enhancing peripheral tissue sensitivity to insulin, improving IR, and inhibiting cholesterol biosynthesis and storage. However, fatty liver can be formed in a model with a high fat diet, liver and muscle glycogen synthesis ability to drop, Therefore, metformin may not significantly improve the pathological features of fatty liver oil red.

Early on, we used the enrichment analysis of key intersection target pathways in network pharmacology to find that inhibition of the AGEs/RAGE/NF-κB signaling pathway could reduce oxidative stress and inflammatory response, reduce tissue damage, and maintain homeostasis of the internal environment. AGEs increase the expression of LCN-2 in many tissues and cells through the RAGE, MAPK, and NF-κB signaling pathways, suggesting that LCN-2 may be a key target molecule for improving IR (57). We aimed to verify that MCDD may regulate NF-κB signal in the inflammatory microenvironment through the target molecule LCN-2 and affect insulin signaling pathway molecules to improve PCOS-IR. When cells are stimulated by high glucose, bacterial lipopolysaccharide, virus, free fatty acids, oxygen free radicals, and cytokines, they can activate IKK upstream kinase and activate IKK, further activate the NF-κB signal pathway, and control the expression of TNF-a, IL-1, IL-2, IL-6, IL-8, PAI-1, MCP-1, some acute phase reactive proteins, and immune receptors, leading to inflammation. Inflammation is usually a physiological response to harmful stimuli, but if clearance fails, a low level of chronic inflammatory response in the body will affect body function (58). Moreover, Insulin can promote the proliferation and differentiation of ovarian cells. In patients with PCOS-IR, insulin tyrosine phosphorylation is weakened and serine phosphorylation is enhanced, thereby weakening the combination of insulin receptor substrate with the upstream protein PI3K of the signal transduction pathway, then inhibiting the activity of cytoplasmic signal transduction pathway and finally induce IR, or on the other hand, IR is induced by genetic mutations in the insulin receptor on tissue membranes, or defects in IRS-1 or glucose transporters. Consequently, our results further proved that MCDD could reduce the contents of serum inflammatory factors and LCN-2 in a dose-dependent manner, implying that it may be related to the NF-κB signaling pathway. MCDD significantly influenced the classical upstream molecules of PI3K-AKT in the insulin signaling pathway, including the expression of *Insr/Irs-1/Glut4* mRNA and the expression of *Lcn-2* gene, and it decreased *Nf-κb* and *Tnf-α* mRNA. More importantly, MCDD may inhibit the expression of NF-κB phospho-p65 protein and LCN-2 protein, consistent with the results reported by Chang, Z.P. et al. (59–61). In the future, the interaction between NF-κB mechanism molecules and LCN-2 needs to be further explored.

This study showed that letrozole combined with a high-fat diet could successfully induce PCOS-IR. MCDD could improve the general pathological index of PCOS-IR rats, ease the level of inflammatory factors *in vivo*, regulate the expression of molecules related to the NF-κB signaling pathway and insulin signaling pathway, and inhibit the expression of LCN-2 molecules. MCDD may exert its efficacy through the molecular mechanism of LCN-2/NF-κB, which provide a theoretical basis for the clinical application of MCDD and development of new therapeutic targets of PCOS-IR.

## Data availability statement

The original contributions presented in the study are included in the article/supplementary material. Further inquiries can be directed to the corresponding author.

## Ethics statement

The protocols involved in this study were in accordance with the rules and guidelines of the Experimental Animal Centre of Guangzhou University of Chinese Medicine and were reviewed and approved by the Guangzhou University of Chinese Medicine Animal Care and Use Ethics Committee (ZYD-2021-215). The experiment was performed following international, national, and institutional animal experiment rules. The rats were handled according to internationally accepted principles for the care and welfare of laboratory animals. All the animals were euthanized by cervical dislocation at the end of the experiment.

## Author contributions

YL conceived, designed and directed this study. SL and YZ drafted the manuscript and contributed to figure preparation. SL, YZ, FY, JG, RZ, YK performed the experiments. SL, YZ, FY, JG, RZ, YK substantial contributed to analysis, interpretation of data. SL, YZ, YK, WM, CZ, YY, RL, LZ, HC all participated in revision of the manuscript. All authors contributed to the article and approved the submitted version.

## Funding

This work was supported by grants from the Natural Science Foundation of Guangdong Province (No. 906107517025), National Natural Science Foundation of China (No. 81804135), and Luo Yuankai Zishen Yutai Pill Young and the Middle-Aged Scientific Research Fund Project of China (No. 20190802).

## Acknowledgments

We thank Lingnan Medical Research Center of Guangzhou University of Chinese Medicine for support.

## Conflict of interest

The authors declare that the research was conducted in the absence of any commercial or financial relationships that could be construed as a potential conflict of interest.

## Publisher’s note

All claims expressed in this article are solely those of the authors and do not necessarily represent those of their affiliated organizations, or those of the publisher, the editors and the reviewers. Any product that may be evaluated in this article, or claim that may be made by its manufacturer, is not guaranteed or endorsed by the publisher.

## Abbreviations

AKT, Protein kinase B; E2, Estradiol; FAI, Free androgen index; FBG, Fasting blood glucose; FINS, Fasting insulin; FSH, Folicle stimulating hormone; GLUT 4, glucose transporter 4; HDL-C, high-density lipoprotein; HE, Hematoxylin-eosin; HOMA-IR, Insulin resistance index; LCN-2, Lipoprotein-2; LDL-C, Low density lipoprotein; IFN-γ, Interferon-γ; IKK, IκB kinase; IL-1, Interleukin-1; IL-6, Interleukin-6; IL-1 β, Interleukin-1β; INSR, insulin receptor; IR, Insulin Resistance; IRS-1, Insulin receptor substrate-1; ISI, Insulin sensitivity index; LH, Luteinizing hormone; MAPK, Mitogen activated protein kinase; MCDD, Modified Cangfu Daotan decoction; Met, Metformin; NGAL, Neutrophil gelatinase-associated Lipocalin; NF-κB, Nuclear transcription factor kappaB; OGTT, Oral glucose tolerance test; PCOS, Polycystic Ovary Syndrome; PI3K, Phosphatidylinositol 3 kinase; SHBG, Sex hormone binding globulin; T, Testosterone; TCHO, Total cholesterol; TG, Triglyceride; TNF-α, Tumor necrosis factor alpha.
